# The perfect carbon TOCSY (iPE TOCSY) facilitates assignment of large biomolecules

**DOI:** 10.1007/s10858-026-00496-z

**Published:** 2026-09-24

**Authors:** Arnelle Löbbert, Aditya Pokharna, Philip Rößler, Simon H. Rüdisser, Alvar D. Gossert

**Affiliations:** 1 Department of Biology, ETH Zürich, Zürich, 8093 Switzerland; 2 Present Address: Novartis Pharma AG, Basel, Switzerland; 3 Present Address: MRC Laboratory of Molecular Biology, Cambridge, UK; 4 Present Address: University of Toronto, Toronto, Canada

**Keywords:** Perfect echo, TOCSY, Large proteins, Homonuclear coupling

## Abstract

Side chain assignments are essential for in-depth characterization of proteins. We present here two refined TOCSY experiments based on the use of a variant of “perfect echo” to partially suppress the negative effects arising from homonuclear ^13^C couplings in the indirect dimension: The iPE-(H)CCH-TOCSY for correlating aliphatic and aromatic side chains and the iPE-(H)C(CCO)NH-TOCSY for linking side chains, in particular methyl groups, to the backbone. The iPE-TOCSY pulse sequences result in spectra of higher sensitivity and higher resolution at the same time compared to widely used standard experiments. Higher sensitivity is achieved by delayed dephasing of the signal due to homonuclear couplings and via the COSY-type C-C transfer inherent in PE-schemes. For a protein like MBP (43 kDa), sensitivity increases of up to 3-fold are shown. For enhancing resolution, the iPE scheme allows prolonging the effectively usable *t*_1_ time from 11 to 16 ms, which leads to sharper lines and reduces overlap significantly. These two experiments have the potential to increase the size limit for side chain assignments and simplify spectral analysis by reducing overlap.

**Supplementary Information:**

The online version contains supplementary material available at https://doi.org/10.1007/s10858-026-00496-z.

## Introduction

NMR spectroscopy provides a plethora of techniques for studying the structure and dynamics of biomolecules. Resonance assignment of these molecules enables research at atomic resolution (Wüthrich 1986). However, the assignment of resonances still remains a bottleneck for the investigation of large systems, which are of growing interest in biological research. The process of resonance assignment is based on triple resonance experiments to assign backbone resonances in a first step, and experiments employing total correlation spectroscopy (TOCSY) to decorate the backbone with side chain resonances in a second step (Cavanagh et al. 1996). In high molecular weight proteins, however, fast transverse relaxation leads to rapid loss of signal in these inherently long 3D experiments, leaving little – if any – signal for analysis. Deuteration of the protein reduces transverse relaxation, such that backbone assignment of proteins up to 50 kDa becomes possible (LeMaster 1989; Yamazaki et al. 1994; Leiting et al. 1998; Goto et al. 1999). For side chain experiments, even with partial deuteration sensitivity remains a challenge (Goto et al. 1999). In this work we therefore aimed at increasing the sensitivity of the side chain assignment experiments.

For assignment of side chain resonances, experiments based on ^13^C-isotropic mixing are typically used due to their superior sensitivity (Shaka et al. 1983, 1988; Braunschweiler and Ernst 1983; Bax and Davis 1985; Bax et al. 1990). The two main strategies are the H*CC*H-TOCSY connecting all side chain resonances, and the HC(*CC*)CONH-TOCSY experiment, linking side chain CH moieties directly to the backbone (nuclei mixed in the TOCSY step are in italics). Several aspects of these experiments have been optimized since their introduction (Hilty et al. 2002; Peti et al. 2004; Gossert et al. 2011; Xia et al. 2020), for instance, they were previously limited by the bandwidth of the spin-lock sequence and required for example separate recording of HCCH-TOCSY spectra for aromatic and aliphatic side chains, respectively. However, with advanced hardware like cryogenically cooled probes or micro coil probes, spin lock fields can be generated that are strong enough to cover the entire necessary bandwidth to mix aliphatic and aromatic moieties in one experiment at fields up to 700 MHz, strongly facilitating the assignment procedure (Kovacs et al. 2005; Ban et al. 2012; Kovacs and Gossert 2014).

In the HC(CC)CONH-TOCSY, on the other hand, a wide bandwidth ^13^C-spin lock can be used to include the C’ in the mixing sequence, thus directly transferring the magnetization from aliphatic to carbonyl carbons, without the need for an extra INEPT transfer from C^α^ to C’. This considerably shortens the pulse sequence by about 10 ms, which is crucial for measurement of large biomolecules, where fast transverse relaxation during the experiment leads to rapid signal loss. In previous studies, we explored the use of up to 17.9 kHz ^13^C-spin lock power (corresponding to a 90° pulse of 14 µs) to circumvent the magnetization transfer by a standard INEPT. Hereby, substantially improved signal intensities for isotropic mixing-based experiments commonly used for side chain assignments such as the 3D (H)C(*CCO*)NH-TOCSY and the carbon-detected 2D (H)CC-TOCSY could be achieved (Kovacs and Gossert 2014).

In the present study, we aimed at further increasing signal intensities of the HC(C)H- and an (H)C(CCO)NH-TOCSY by overcoming the limitations imposed by homonuclear ^1^*J*_CC_ couplings during the ^13^C chemical shift evolution (*t*_1_) period. In uniformly ^13^C-labeled proteins, the *t*_1_ duration is limited because signals evolve into antiphase under the influence of ^1^*J*_CC_ (~ 36 Hz), effectively leading to zero detectable signal after 13 ms of evolution. Therefore, the ^13^C chemical shift is typically only sampled up to about 6–9 ms. We explored the potential of a perfect echo (PE) element in order to at least partially refocus ^1^*J*_CC_ couplings and therefore enable longer *t*_1_ times, resulting in sharper and more intense signals. The perfect echo was first described independently by Takegoshi et al. (1989) and van Zijl et al. (1990) aiming at the suppression of homonuclear couplings in ^1^H spin echoes. Behind the term perfect echo stands the addition of a 90° pulse at the center of a double spin echo to refocus *J* modulation of a weakly-coupled two-spin system AX. Notably, Aguilar et al. extended the applicability of the perfect echo to general spin systems, showing that for short spin echo durations 2τ (as in τ−180°–τ), anti-phase terms from multiple scalar couplings *J* are refocused, provided that the condition sin(2π*J*_ij_2τ) < < 1 is met (Aguilar et al. 2012). In the following years, the perfect echo became a commonly used element employed in 1D as well as 2D ^1^H NMR experiments to reduce the signal distortions due to homonuclear couplings arising in a standard spin echo (Aguilar et al. 2012; Kaltschnee et al. 2014; Koos et al. 2016; Nolis and Parella 2018; Sinnaeve 2018; Parella 2019).

Here, we aim at extending the scope of application of the perfect echo to refocus homonuclear ^1^*J*_CC_ couplings in the *indirect* dimension of higher dimensional experiments (hence iPE, for PE in the indirect dimension). One main difference to the well-established case of *J*_HH_ is that ^1^*J*-couplings between ^13^C nuclei are much stronger (~ 35–55 Hz) such that the maximal delay 2τ for which *J*-couplings can be refocused is rather short (< 3 ms). The other important difference is that, in the indirect dimension, it is advantageous to implement only half of the perfect echo, such that *J*-coupling refocusing occurs a few milliseconds into the evolution period rather than at its beginning. This allows prolonging the sampling period by ~ 50%. Based on these considerations, we implemented a variant of the perfect echo scheme in combination with a wide bandwidth ^13^C-spin lock into two commonly used 3D experiments for side chain assignment: the HC(C)H- and an (H)C(CCO)NH-TOCSY. We explored parameters like the optimal perfect echo delay, the maximal *t*_1_ sampled, and TOCSY mixing times. In general, a significant improvement in sensitivity can be achieved with the perfect echo scheme and for large biomolecules (e.g. MBP, 43 kDa), up to 3-fold signal enhancement was attained, facilitating the assignment of biologically highly relevant proteins.

## Materials and methods

### Protein samples

Spectra were acquired on 3.8 mM U-^13^C,^15^N-TM1290 (protein 1290 from *Thermatoga maritima*, 13 kDa) in 20 mM sodium phosphate at pH 6.0 and 2 mM NaN_3_ (Etezady-Esfarjani et al. 2003), 0.6 mM MBP (maltodextrin binding protein, 43 kDa) in 20 mM sodium phosphate at pH 6.8 and 50 mM NaCl. MBP was ^2^H,^13^C,^15^N-labelled with specific protonation of methyl groups of leucine, valine and isoleucine (δ_1_-methyl only) (Goto et al. 1999).

Further, 1 mM U-^13^C,^15^N-Nb80 (nanobody 80, 14 kDa) in 20 mM HEPES (pH 7.5), 300 mM NaCl (Manglik et al. 2017) as well as 0.5 mM U-^13^C,^15^N-GB1 (B1 domain of protein G, 8 kDa) in 20 mM HEPES (pH 7.5), 300 mM NaCl were prepared following previously described to standard procedures (Rößler et al. 2020b).

The sample of 0.8 mM U-^13^C,^15^N-labelled OB domain protein (oligonucleotide binding domain of ComEC from *Moorella glycerini*, 14 kDa) in 25 mM NaPi (pH 7.2), 50 mM NaCl was kindly provided by Matthew Stedman (Stedman et al. 2025).

### NMR spectroscopy

Spectra were recorded on a Bruker Avance Neo 500 and 600 MHz spectrometer equipped with a CP-QCI 1H&19F/31P-13C/15N/2H Z-Grad and CP-TCI 1H&19F-13C/15N-2 H ZGrad probe head, respectively. Sample temperature was set to 298.1 K for all measurements, except of the measurement on MBP. Here, temperature was increased to 310 K. The parameters of the developed experiments as well as for the reference experiment are summarized below. Details of the optimization process of individual parameters can be found in the main text.

In the iPE-HC(C)H-TOCSY experiment, the iPE-delay was set to 2.3 ms. The FLOPSY16 mixing time for the iPE and the reference experiment was set to 20 ms with a spin lock power of 13.9 kHz at 500 MHz and 16.7 kHz at 600 MHz. For the ^13^C–^13^C-spin lock covering aliphatic and aromatic ^13^C-frequencies (bandwidth ca. 140 ppm), the ^13^C-offset was set to 75 ppm in order to center it with respect to the chemical shifts of the C^γ^–C^β^ pair of spins.

Parameters for iPE-(H)C(CCO)NH-TOCSY were chosen as follows: The iPE-delay was set to 2.3 ms. The FLOPSY-16 mixing time was set to 23 ms and a spin lock field strength of 16.7 kHz was applied. Measurements were conducted on a 500 MHz spectrometer. The ^13^C-carrier frequency was set to 18 ppm during ^13^C-evolution moved to 95 ppm for the ^13^C-spin lock and to 173 ppm for the transfer from carbonyl to amide nitrogen. For reference experiments, the standard BRUKER pulse sequence (hccconhgpwg3d3) was chosen. DIPSI mixing time was set to 23 ms at a spin lock field strength of 16.7 kHz. Offset parameters were set to the same values as for the modified (H)C(CCO)NH-TOCSY.

## Results and Discussion

### ^13^C perfect echo in an ^1^H,^13^C refocused INEPT experiment

**Fig. 1:**
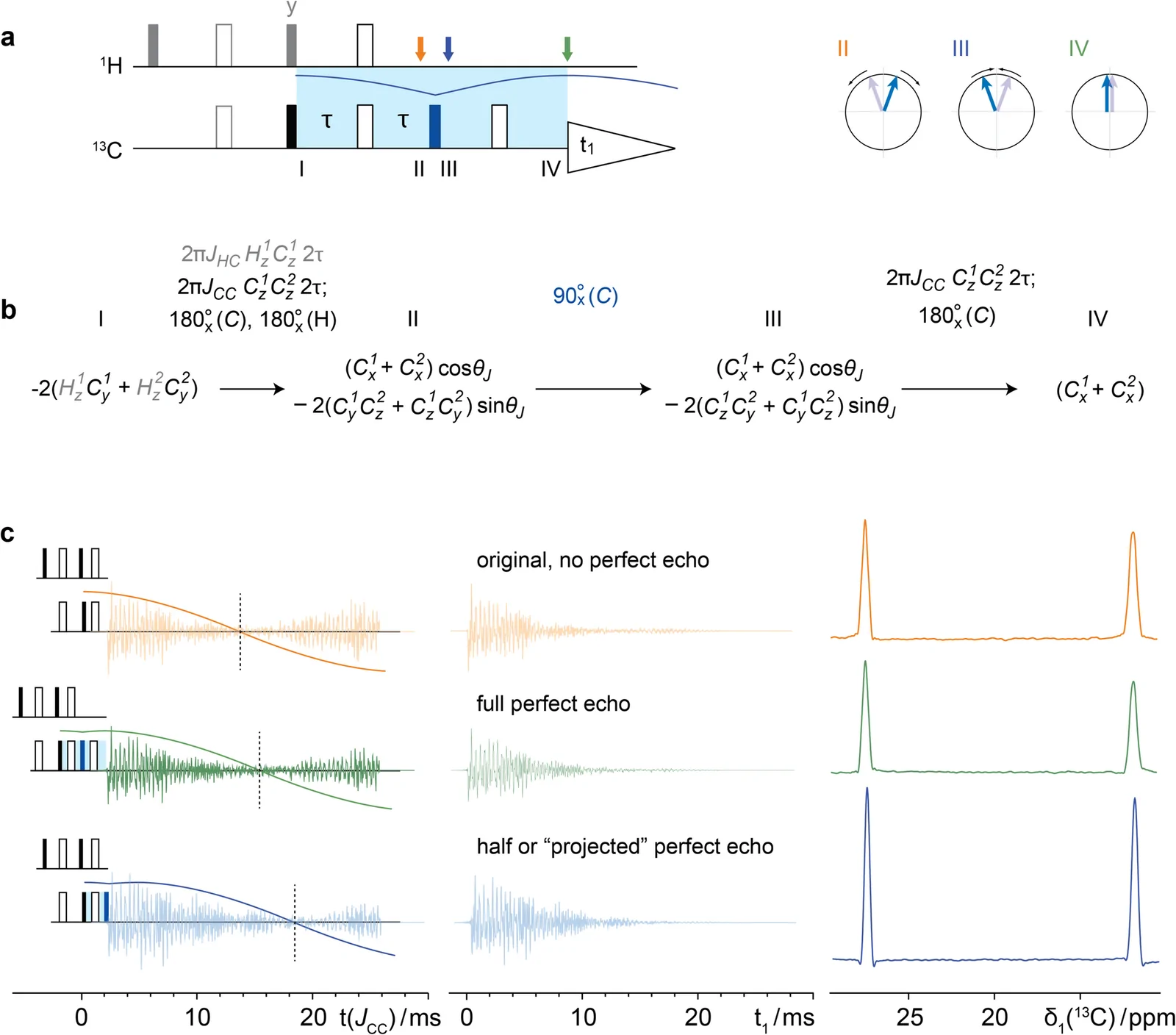
Benefits of a perfect echo element in the carbon dimension of a refocused INEPT sequence. (**a**) Pulse sequence of the perfect echo element in the context of a ^13^C detected refocused ^1^H,^13^C-INEPT. Vertical bars and empty rectangles represent rectangular 90° and 180° pulses, respectively. The perfect echo element is shown with blue background and the 90° pulse at the midpoint of the double spin echo, refocusing the homonuclear ^1^*J*_CC_ coupling, is highlighted in blue. The magnitude of observable in-phase ^13^C magnetization under ^1^*J*_CC_ coupling is shown as a blue line with an exaggerated coupling constant for clarity. The colored arrows indicate the start of the evolution period of the respective schemes in (c), along with a vector representation on the right. (**b**) Product operator formalism representing the perfect echo element, where for simplicity θ_*J*_ = 2π*J*_cc_2τ. The coupling to ^1^H is considered during the refocusing INEPT, but omitted in the second echo, as it has no net effect. (**c**) Simplified pulse schemes with resulting FIDs (left), FIDs after apodization (middle) and 1D spectra (right) are depicted for β and γ resonances of u-^13^C labeled leucine. From top to bottom: original 1D ^13^C sequence (orange), a control experiment including the full perfect echo element (green), and a shortened version with the first half of the perfect echo element (blue) where detection is started directly after the 90° pulse (blue pulse), without a second spin echo. The perfect echo leads to a delayed *t*_1_ zero crossing represented by the dashed lines. This allows prolonged signal recording as can be seen in the FID

The perfect echo has been shown to be a useful scheme to avoid signal distortions produced by the evolution due to homonuclear *J*_HH_ couplings. In order to examine the possibility to extend the scope of the perfect echo to suppress *J*_CC_ couplings in 3D assignment experiments, we first implemented the PE element in a ^13^C-detetcted 1D experiment (Fig. 1). In a PE element, a 90° pulse is applied at the center of a double spin echo with a phase orthogonal to the preceding 90° pulse (for example: 90_x_-τ−180_x_-τ−90_**y**_–τ−180_x_–τ). This leaves the in-phase I_y_ and S_y_ terms unaffected but exchanges the antiphase terms I_x_S_z_ and S_x_I_z_, leading to a change of their signs and therefore reversing the net effect of the *J* modulation (Fig. 1b) (Aguilar et al. 2012). In the case of a refocused ^1^H,^13^C INEPT experiment, as used at the start of a TOCSY experiment for instance, the situation is slightly different: here, one starts with antiphase magnetization on ^13^C with respect to ^1^H (−2C_y_H_z_), which is refocused to in-phase magnetization during the refocusing INEPT step, leading to in-phase magnetization – but along x (C_x_). Thus, the perfect echo pulse needs to be applied along the x-axis (Fig. 1a and b).

First, a spectrum using the original pulse sequence for a refocused ^1^H,^13^C INEPT was recorded on a sample of u-^13^C leucine. (Fig. 1c, orange). Here, the detectable FID has a zero crossing due to ^1^*J*_CC_ coupling at 13 ms after the first 90° pulse on carbon. Since the signal is only recorded after the refocusing delay of 2.3 ms, the effective *t*_1_ is 10.7 ms. In a next step, the full PE element including a second spin echo was used. The incorporation of the PE leads to a delayed dephasing of magnetization due to ^1^*J*_CC_-coupling, delaying the zero crossing in *t*_1_ (~ 13 ms vs. ~10.7 ms), and allowing prolonged signal recording (Fig. 1c, green). However, the resulting spectrum did not show a significant signal increase compared to the original pulse sequence. Therefore, a shortened version of the PE was tested, starting acquisition right after the perfect echo 90° pulse which reverses the homonuclear ^1^*J*_CC_ couplings (Fig. 1c, blue). Hereby, the *t*_1_ zero crossing can be delayed even further to ~ 15.3 ms since the time point of *J*_CC_-refocusing is projected into the evolution time. This leads to a signal increase of ~ 50%. The signal increase can be attributed to two main factors: Increased resolution leads to a narrower peak with higher intensity, and the initially raising FID further leads to narrowing of the peak shape. This effect is similar to that of delayed decoupling schemes and reminiscent of resolution-enhancing window functions, but without the added noise level of the latter (Cavanagh et al. 1996; Rößler et al. 2020a).

### Optimization of perfect echo delay and sampling time in the indirect ^13^C dimension

**Fig. 2:**
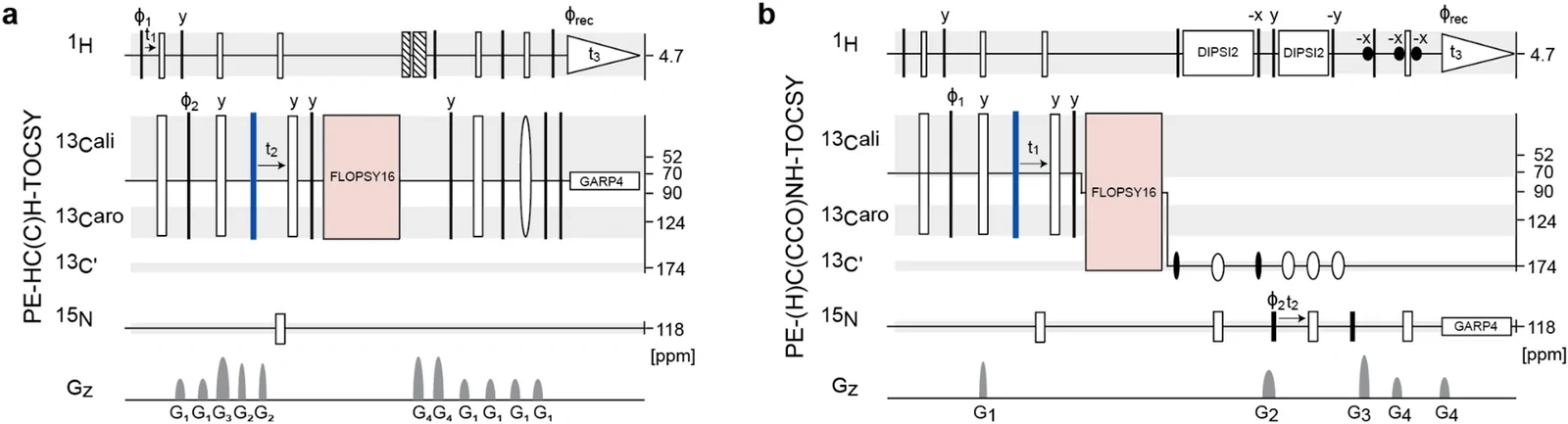
Pulse sequence of the iPE-HC(C)H-TOCSY (**a**) and the iPE-(H)C(CCO)NH-TOCSY (**b**). Vertical bars and empty rectangles represent rectangular 90° and 180° pulses, respectively and their phases are indicated above the pulses if they differ from x (= 0°). Isotropic mixing sequences or decoupling sequences, are represented by rectangles including their names (Shaka et al. 1985, 1988; Kadkhodaie et al. 1991) and non-rectangular pulses are depicted in elliptical shape. The intended excitation bandwidth of pulses is represented by their vertical extension. The grey shaded horizontal rectangles represent frequency bands of ^1^H, ^15^N and ^13^C nuclei, with the ^13^C bandwidth being subdivided into regions of aliphatic (^13^C^ali^), aromatic (^13^C^aro^) and carbonyl (^13^C’) resonances. The continuous lines on which pulses are centered depict the carrier frequency of each rf-channel, which in the case of ^13^C is moved several times during the pulse program. Selected ppm values are given on the right. Quadrature detection in indirect dimensions was obtained using the States-TPPI method. Pulsed field gradients along the z-axis (G_z_) consist of smoothed square shapes of 1.0ms duration and are represented by grey shapes. Relative strengths for G_1_:G_2_:G_3_:G_4_ are 16:16:30:60 for the HCCH and 50:40:60:30 for the HCCCONH-TOCSY, respectively

As the implementation of the iPE element was shown to be highly beneficial for the 1D ^13^C experiment, the next step was to set up the 3D experiments required for resonance assignment. As described earlier, for the 3D experiments, we aimed at combining the advantages of the iPE with high-power mixing pulses to further enhance the sensitivity of the pulse sequence (Fig. 2). In the following section, the optimization of several parameters including the iPE delay, acquisition time in the indirect dimension, and TOCSY mixing time will be discussed.

In a first step, the iPE delay was subjected to optimization. We define here the period of 2τ of the spin echo during which *J*_CC_ coupling is active as the iPE delay (see Fig. 1a), as it is a crucial parameter for this experiment. In principle, longer iPE delays would increase *t*_1_ before the first zero crossing by two times the iPE delay. As this holds the potential of increased signal intensities, iPE delays ranging from 2.3 to 4 ms were tested using a test sample of a 3.8 mM protein TM1290 from *Thermatoga maritima*. The minimal delay of 2.3 ms is the weighted time to simultaneously refocus the antiphase − 2H_z_C_y_ magnetization of CH, CH_2_ and CH_3_ groups (Burum and Ernst 1980), and the maximal delay of 4 ms is where sinθ_*J*_ reaches a value of 0.45, clearly exceeding the perfect echo condition for spin systems of orders higher than 2. The iPE delay can either be dynamically increased with increasing *t*_1_, or kept static for all values of *t*_1_. Overall, no significant benefit from longer iPE delays was observed, neither in the dynamic nor in the static case (Figure S1). Therefore, a static iPE delay of 2.3 ms was chosen for simplicity.

As the implementation of the perfect echo enables prolonged signal recording due to a delayed *t*_1_ zero crossing caused by homonuclear C-C couplings, *t*_1_ acquisition times between 7.9 and 15.9 ms were evaluated. Results were analyzed regarding two different objectives: increased signal-to-noise ratio (S/N) and enhanced resolution.

**Fig. 3:**
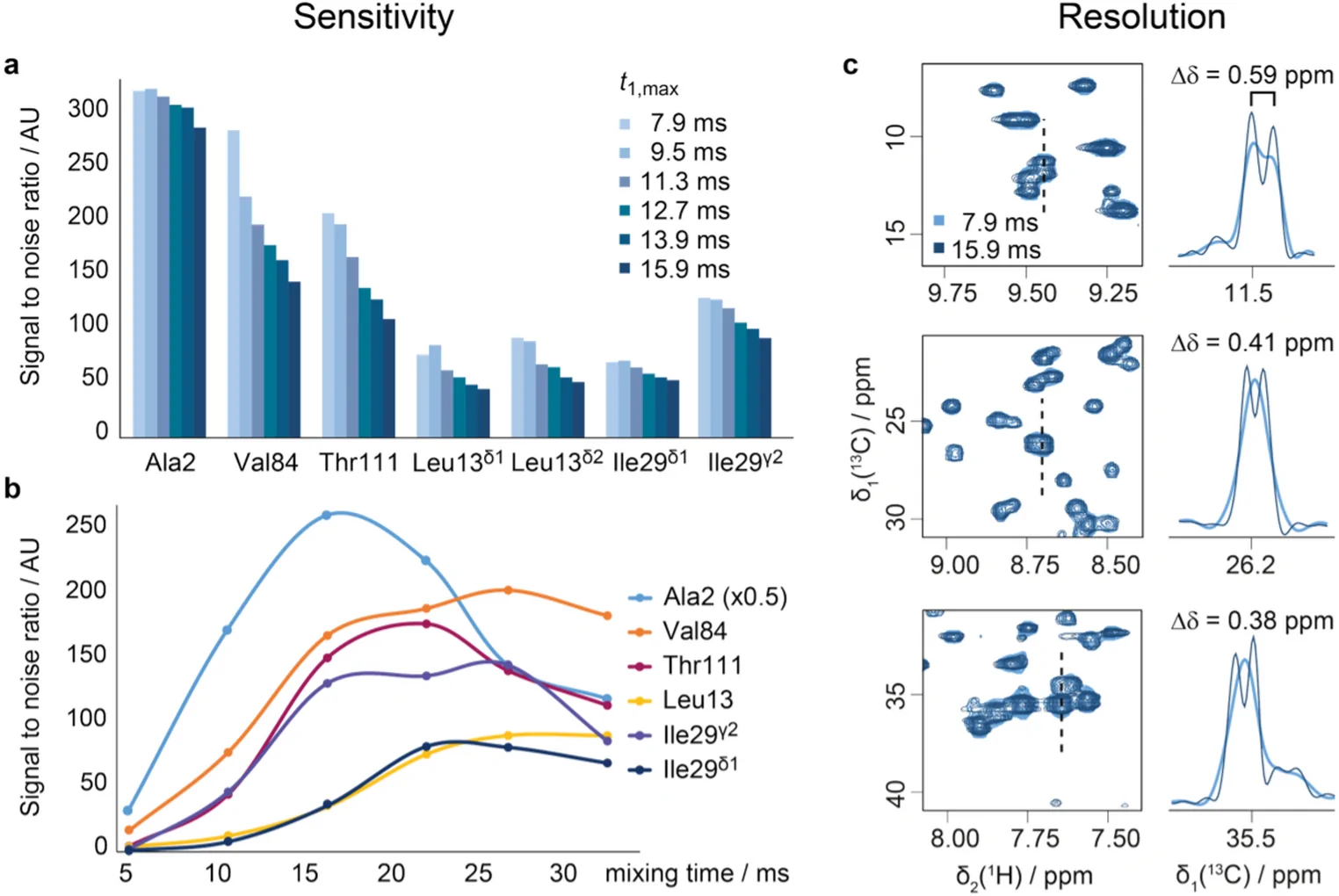
Optimization of TOCSY parameters for sensitivity or resolution. Measurements were conducted on a 3.8 mM TM1290 sample (**a**,** c**) and 0.8 mM OB sample (**b**). Data of the iPE-(H)C(CCO)NH-TOCSY experiment is shown. For all experiments in this figure, the iPE delay was set to 2.3 ms. The static field was 500 MHz and a spin lock power of 16.7 kHz was applied. (**a**) Different acquisition times *t*_1_ (7.9–15.9 ms) were examined for one representative of all amino acids carrying a methyl group. The optimal acquisition time was evaluated based on the signal-to-noise ratio of the individual signals and values were normalized per unit of time $(S/N*\sqrt{\frac{{TD}_{max}}{{TD}_{eff}}})$ i.e. an experiment with fewer *t*_1_ increments allows for more scans in a given time.) A mixing time of 27 ms was used in these experiments. (**b**) Different acquisition times in the ^13^C dimension (*t*_1,max_ = 7.9–15.9 ms) were evaluated regarding their resolution using the OB domain as a test protein. Overlays of the 2D plane of a iPE-HC(C)H-TOCSY of the shortest (*t*_1,max_ = 7.9 ms, light blue) and longest (*t*_1,max_ = 15.9 ms, dark blue) tested *t*_1,max_ values is depicted for three representative regions. 1D slices are shown for selected overlapping peaks indicated by the dashed line in the 2D spectra, demonstrating that iPE allows to separate otherwise overlapping signals. (**c**) Experimental TOCSY transfer curves of the methyl groups of respective amino acids. The signal to noise ratio of ^13^C^Methyl^-^1^H^N^ cross peaks is plotted against the mixing times to assess the peak magnetization transfer for the individual TM1290 signals (13 kDa). Acquisition time *t*_1_ was set to 15.9 ms

Within the first analysis, results were based on the S/N of one representative of all amino acids carrying a methyl group in the protein TM1290 (Fig. 3b). The fact that a lower number of increments allows for a higher number of scans in the same experiment time was taken into account. The normalized results show a general tendency towards decreased S/N with longer recording of the indirect carbon dimension. For Val84, Thr111, Leu13_δ2_ and Ile29_γ2_ an acquisition time of 7.9 ms gives the highest S/N, whereas for Ala2, Leu13_δ1_ and Ile_δ1_ highest values can be obtained by recording the carbon dimension for 9.5 ms. Similarly to the extended iPE delays, it seems that prolonged recording of the signal does not result in an increase of the signal intensity. In our experiment, for some amino acids the use of *t*_1,max_ = 9.5 ms resulted in slightly increased S/N values compared to *t*_1,max_ = 7.9 ms. Since the increase was only minor and the effect of *T*_2_ relaxation is expected to be stronger for larger biomolecules, a *t*_1_ of 7.9 ms should be applied for optimized S/N.

In contrast, enhanced resolution is obtained with longer acquisition times *t*_1_ as demonstrated in Fig. 3b. In particular, for aliphatic residues, prolonged signal recording of the carbon dimension, enabled by of homonuclear *J*_CC_ couplings, results in substantially improved peak resolution. While an acquisition time of 7.9 ms leads to hardly any discrimination of the two observed peaks, signal recording of 15.9 ms allows clear separation of the previously overlapped signals. Even for aromatic residues, which experience strong couplings, a positive effect on the resolution becomes visible (Fig. 5c). Depending on the aim of the individual study (resolution vs. sensitivity), acquisition times should be adjusted accordingly.

### Adjusting high-power spin lock in perfect echo experiments

One aspect of the perfect echo element was not yet mentioned: the central perfect echo 90° pulse acts as a magnetization transfer element, similar as in a COSY experiment (C^1^_x_C^2^_z_ magnetization is converted into C^1^_z_C^2^_x_). Thus, some magnetization transfer already happens before the TOCSY spin lock, potentially allowing to shorten the spin lock element. The spin lock time was therefore subjected to optimization.

In standard experiments, a spin lock field strength of 10–11 kHz is commonly applied. However, nowadays, cryogenic probes allow the use of 1.5-fold higher γB_1_ strengths. Mixing times ranging from 5.7 to 33.2 ms (5.7, 11.4, 17.1, 22.8, 27.5, and 33.2 ms) were tested applying 16.7 kHz spin lock power on a 500 MHz spectrometer. The signal to noise ratio (S/N) of one representative of each amino acid carrying a methyl group is plotted as a function of mixing times in Fig. 3c. Build-up curves for the cross-peak intensities display first maxima for Ala and Ile_γ2_ at 17 ms, whereas for most of the other cross peaks a mixing time of 22.8 ms seems to be most efficient. For Val, the maximum transfer is achieved at 27.5 ms. In principle, transfers over a larger number of bonds usually require longer mixing times to maximize the magnetization transfer. Given the heterogeneous length and branching of the carbon chain in individual amino acids, a compromise regarding the applied mixing time is needed to achieve an optimal transfer of the magnetization. If a measurement should cover all aliphatic amino acids, our results suggest using a mixing time of 22.8 ms. Further, the plot shows that mixing times beyond 22.8 ms do not substantially increase the peak intensities for any of the tested methyl groups. It is to mention that if a specific labeling pattern is used (e.g. Alanine-only), this parameter should be adapted accordingly.

As high-power mixing pulses open up the possibility to measure aliphatic and aromatic side chains in one experiment, mixing times for aromatic side chains also have to be taken into consideration. Previously, maximized transfers between 20 and 25 ms depending on the aromatic system were reported (Kovacs and Gossert 2014). For example, a transfer time around 23 ms was shown to be most suitable to transfer the magnetization from aromatic carbons the C_α_ in phenylalanine. Hence, the suggested mixing time of ~ 23 ms is a well-suited compromise to obtain highest S/N for aliphatic as well as aromatic side chains.

### Evaluation of modified experiments on different protein samples

In a next step, we aimed at evaluating the benefits of the newly included elements into the TOCSY experiments. To this end we compared the signal intensities obtained by measuring a reference experiment (BRUKER standard pulse sequence hccconhgpwg3d3), a (H)C(CCO)NH-TOCSY experiment containing high-power mixing pulses only, and the iPE-(H)C(CCO)NH-TOCSY experiment containing both, high-power mixing pulses, as well as the iPE element. Note that in the latter two experiments the C^α^-C’ INEPT is removed, as the TOCSY transfer includes C’.

**Fig. 4:**
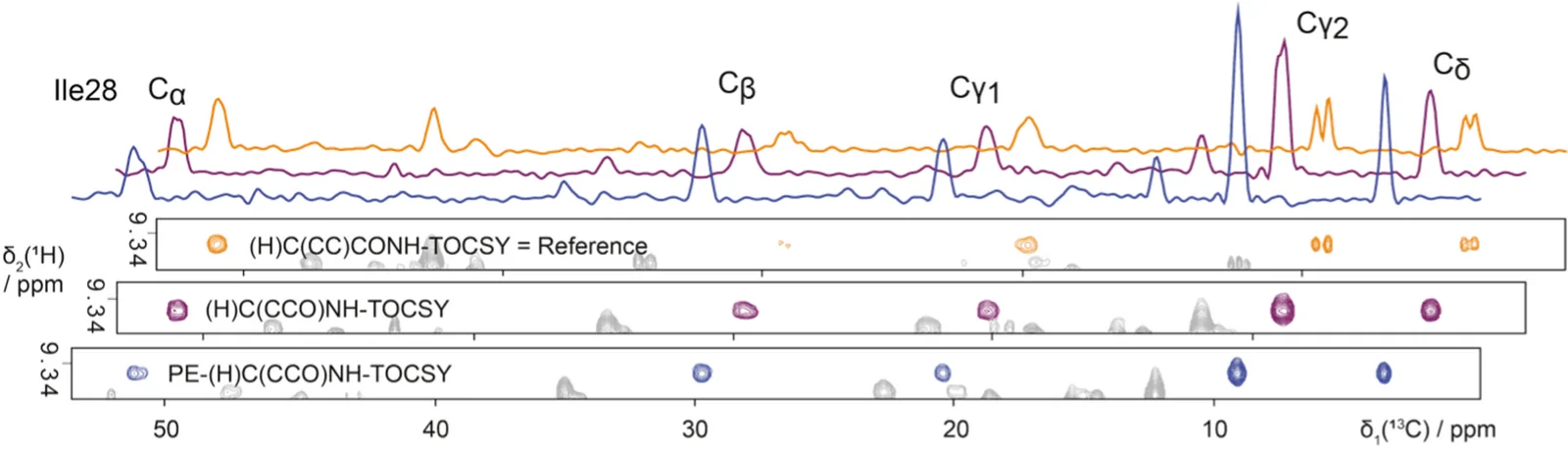
Comparison of different TOCSY variants shows sensitivity improvement by iPE-(H)C(CCO)NH-TOCSY. 1D slices (top) of the respective 2D [^1^H,^13^C] planes (bottom) of TM1290 Ile28 are shown for the reference TOCSY (orange), the (H)C(CCO)NH-TOCSY (purple) and the iPE-(H)C(CCO)NH-TOCSY (blue). Spectra were recorded for ~ 1.5 h on a 500 MHz spectrometer. Acquisition time *t*_1_ was set to 15.9 ms for maximized resolution. The iPE delay was set to 2.3 ms and the mixing time was 27 ms at 16.7 kHz spin lock field strength

Results show a remarkable signal increase for both high-power implementations, exemplified on the signals of Ile28 of TM1290 (Fig. 4). The use of high-power mixing pulses (Fig. 4, purple) compared to the reference TOCSY (Fig. 4, orange) leads to a substantial signal increase. The additional implementation of the iPE element leads to clearly sharper lines, resulting in an additional signal increase compared to the two other TOCSY variants, up to 1.4 and 3-fold compared to the high-power and standard experiments, respectively.

Theoretically, high-power mixing pulses should lead to a signal increase based on the overall shortened pulse sequence, as previously described. Interestingly, here, not only an increase of the signals is observed but for some residues, e.g. Ile28 C_γ2_, also the splitting of the signals is reduced. We’re unclear about the source of this effect and currently attribute this to an imperfect spin lock which allows certain couplings to further evolve. Despite the reduction of signal splitting in the carbon dimension, the signals remain broad due to incomplete of the homonuclear ^1^*J*_CC_ couplings. With the implementation of the iPE scheme, this effect can be minimized, leading to sharp singlets with substantially increased intensities. When taking a closer look at the individual residues, it becomes clear, that the terminal methyl groups Ile28 C_γ2_ and Ile28 C_δ_ benefit the most from perfect echo, whereas the gain for Ile28 C_α_ is only minor. The two methyl groups of isoleucine come closest to an isolated two-spin system, such that the iPE element is most effective here. Ile28 C_β_ has three neighboring carbons, i.e. it is a higher order spin system. For optimal refocusing of homonuclear couplings, the condition of sinθ_*J*_ < < 1 must be fulfilled. Here, sinθ_*J*_ has a value of 0.25, which is at the limit. For comparison: in homonuclear ^1^H experiments signal reduction is evident starting with perfect echo delays of 5 ms, representing sinθ_*J*_ ≈ 0.1. Nevertheless, signal splitting can be significantly reduced for the Ile28 C_β_. In contrast, C_α_ has additional couplings to the carbonyl with ^1^*J*_CαC’_ ≈ 55 Hz, which lead to sinθ_*J*_ values close to 0.5, that are clearly beyond the general condition of PE refocusing. Carbonyls can be decoupled with selective pulses, but in this case, we gave higher priority to include aromatic signals in the HCCH-TOCSY, which was incompatible with carbonyl decoupling by shaped pulses, as they affect aromatic signals.

As a last optimization step, we evaluated a DIPSI vs. a FLOPSY type mixing scheme for the ^13^C-^13^C TOCSY transfer (Figure S2). With the latter scheme, enhancements around 10% were obtained and therefore the FLOSPY mixing scheme was used for further experiments.

### Improved sensitivity and resolution of iPE-HC(C)H-TOCSY and iPE-(H)C(CCO)NH-TOCSY with proteins of different sizes

To illustrate the potential of the modified experiments, we performed test experiments on different biological molecules, namely U-^13^C,^15^N labeled GB1 (8 kDa), Nb80 (14 kDa), and OB (14 kDa) for the HC(C)H-TOCSY and U-^2^H,^13^C,^15^N (I, L,V-methyl-^1^H)-labeled MBP (43 kDa) for the (H)C(CCO)NH-TOCSY.

**Fig. 5:**
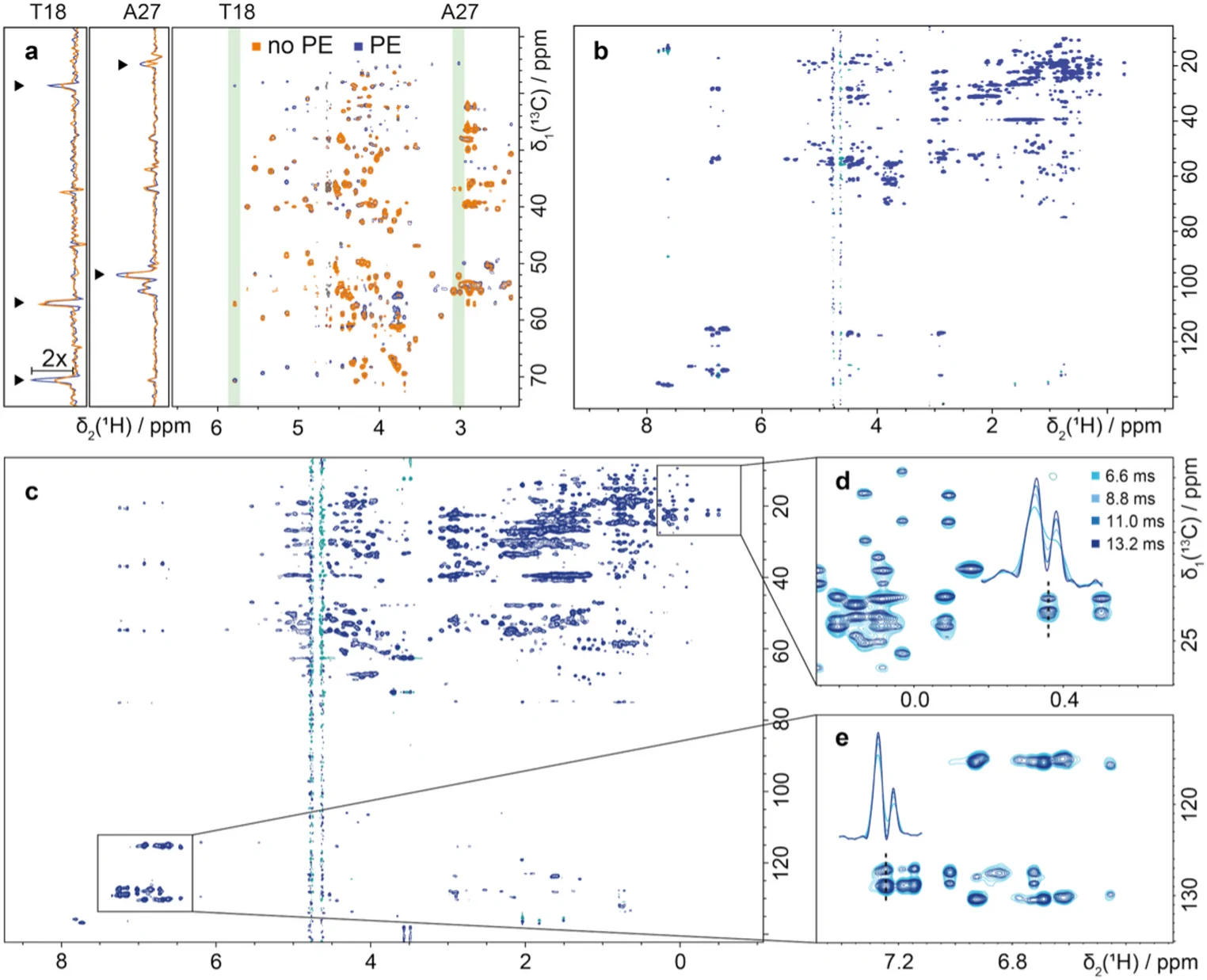
Application of the iPE-HC(C)H-TOCSY to different proteins. (**a**) GB1 protein: 2D [^1^H^N^, ^13^C-methyl]-planes of the reference (orange) and modified (blue) HC(C)H-TOCSY. 1D sliced of highlighted (green) TOCSY towers of T18 and A27 are shown on the left. The iPE-HC(C)H-TOCSY shows up to two-fold signal increase compared to the unmodified counterpart. The spectra were recorded on a 0.5 mM U-^13^C,^15^N labeled GB1 sample over 20 h on a 500 MHz spectrometer. Acquisition time *t*_1_ was set to 15.9 ms. The iPE delay was set to 2.3 ms and the mixing time was 20 ms at 13.9 kHz spin lock field strength. During the spin lock, the ^13^C-carrier frequency was 75 ppm. (**b**) Nanobody 80: 2D [^1^H, ^13^C]-plane of U-^13^C,^15^N labeled Nb80 demonstrates the visibility of aromatic side chains. The spectrum was recorded in ~ 58 h on a 600 MHz spectrometer. Acquisition time *t*_1_ was set to 15.9 ms. The iPE delay was set to 2.3 ms and the mixing time was 20 ms at 16.7 kHz spin lock field strength. The ^13^C-carrier frequency during the spin lock was set to 75 ppm. (**c–e**) OB domain: different acquisition times were evaluated regarding their resolution (*t*_1,max_ = 6.6–13.2 ms). An overview of the spectrum of OB is shown in (c). A representative region is shown for aliphatic (d) and aromatic residues (e), respectively. An overlay of the 2D plane of the shortest (light blue) and longest (dark blue) tested TD is depicted. 1D slices of representative peaks are shown for acquisition times *t*_1_ ranging from 6.6–13.2 ms. It appears that for aliphatic carbons the resolution enhancement is more significant than for aromatic ones, which have strong ^1^*J*_CC_ couplings (~ 56 Hz)

Measurements of U-^13^C,^15^N labeled GB1 using the iPE-HC(C)H-TOCSY compared to the non-iPE counterpart showed a general increase of signal intensities, as more signals become visible in the iPE-HC(C)H-TOCSY (Fig. 5a). This is verified by examining the 1D slices of individual residues (shown for T18 and A27). For T18, a 2-fold signal increase was observed.

Besides shortening the pulse sequence, the use of high-power mixing pulses further enables the simultaneous detection of the aromatic and aliphatic side chains. This is exemplified on U-^13^C,^15^N labeled Nb80 (Fig. 5b) (Manglik et al. 2017). The now observable correlation between aromatic and aliphatic side chains can significantly facilitate the assignment procedure. On U-^13^C,^15^N labeled OB domain (Stedman et al. 2025), the same experiment was recorded to analyze the enhanced resolution (Figs. 3b and 5c). Interestingly, the enhancement on aliphatic side chains is more significant than on aromatic ones. This is most likely due to the stronger ^1^*J*_CC_ couplings found in aromatics than in aliphatics, with values around 56 and 36 Hz, respectively. Therefore, for aromatics the resolution is limited by the faster dephasing, and in the best case, the zero crossing can be extended from 9 to 13 ms with a 2 ms iPE-delay.

**Fig. 6:**
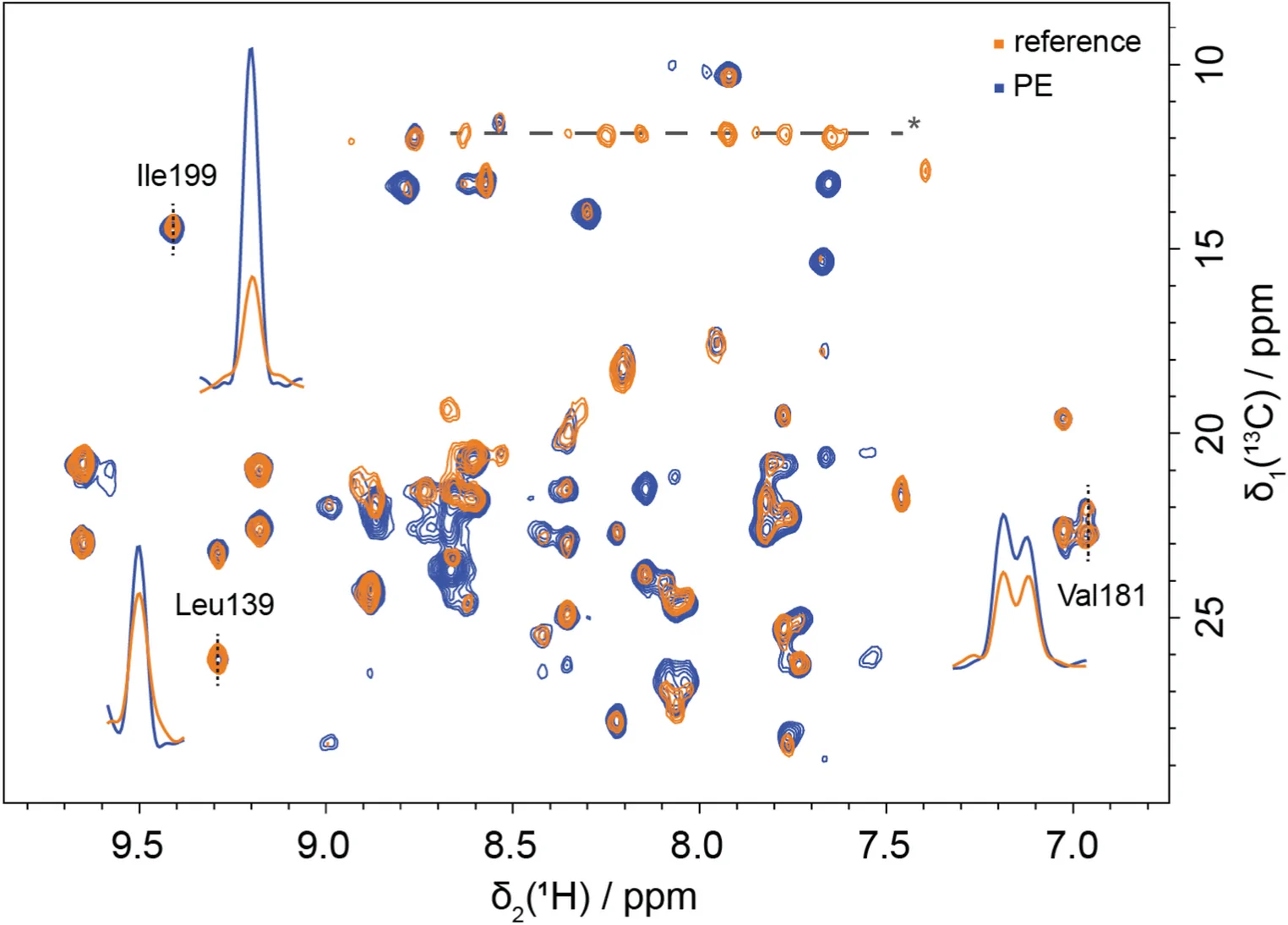
Sensitivity improvement of the modified iPE-(H)C(CCO)NH-TOCSY compared to reference experiment. 2D [^13^C^methyl^, ^1^H^N^]-planes of the standard (orange contours) and modified (blue contours) experiment, respectively. The experiments were measured on a 0.6 mM U-^2^H, ^13^C, ^15^N (I, L,V-methyl-^1^H)-labeled MBP sample. Slices of one representative of each protonated amino acid are shown as inserts in the spectrum. Signals marked with the dashed line and an asterisk (*) are artefacts appearing the reference spectrum. For all three residues the iPE-(H)C(CCO)NH-TOCSY shows superior performance compared to the conventional experiment. Best results are obtained for Ile199 (3-fold enhancement). Spectra were recorded in ~ 20 h each, on a 500 MHz spectrometer. Acquisition time *t*_1_ was set to 7.9 ms. The mixing time was set to 23 ms for the modified (FLOPSY-16) and for the standard experiment (DIPSI). A spin lock field strength of 16.7 kHz was applied in both experiments at 95 ppm. The ^13^C-carrier frequency was set to 18 ppm during ^13^C-evolution moved to the aforementioned values for the spin lock and to 173 ppm for the transfer from carbonyl to amide nitrogen

Assignments of proteins > 30 kDa are commonly reduced to methyl groups of specific amino acids as the higher complexity of the spectra results in increasing signal overlap. The (H)C(CCO)NH-TOCSY correlates resonances of methyl groups with those of the backbone amide group of the following residue, enabling the assignment of the desired methyl groups. In order to demonstrate the usefulness of the iPE-(H)C(CCO)NH-TOCSY, U-^2^H, ^13^C, ^15^N (I, L,V-methyl-^1^H)-labeled MBP was subjected to measurements with and without the two adjustments (iPE element and high-power mixing pulses, Fig. 6). Similarly to the HC(C)H-TOCSY experiments, more signals are visible in the iPE-experiment, indicating a significant signal increase. A closer look at one representative of each of the protonated methyl group containing amino acid (Ile, Leu, Val) was taken by examining their 1D slices across the carbon dimension. Here, it becomes apparent, that the modified experiment gives best results for Ile199 (3-fold signal enhancement), followed by Val181 (1.4-fold signal enhancement) and Leu139 (1.3-fold signal enhancement). The enhancements are a combination of higher signal intensity due to the refocused *J* coupling, the magnetization transfer taking place during the iPE element and the overall shortening of the pulse sequence using the high-power mixing pulses.

## Conclusions

While the perfect echo is routinely used for the suppression of signal distortions due to *J*_HH_ couplings, we demonstrate here an application of a variant of the perfect echo to suppress signal losses due to homonuclear *J*_CC_ couplings. *J*_CC_ couplings are several-fold stronger than *J*_HH_ couplings, such that shorter PE delays are required. Despite this challenge, we could show that when implementing iPE to delay the dephasing of in-phase magnetization in the indirect carbon dimension, signal intensities are improved significantly, with the premise that the effectiveness can vary among different residues. A static iPE-delay of 2.3 ms was shown to be most beneficial. Highest signal-to-noise ratios were obtained with an acquisition time of 7.9 ms in the indirect dimension, while resolution can be substantially enhanced with prolonged acquisition times of up to 16 ms.

We focused on the application of iPE in side chain assignment experiments, where there still is a great lack of sensitivity. We previously showed that high-power mixing pulses can significantly increase the sensitivity and the information content of the HC(C)H- and (H)C(CCO)NH-TOCSY experiments. The successful implementation of iPE in these experiments leads to up to 2–3-fold signal enhancement, *via* combined effects of signal narrowing, magnetization transfer during the *t*_1_ period, and a shortened pulse sequence. This opens up new perspectives for side chain assignment of large proteins and could also be beneficial in other experiments based on in-phase carbon magnetization like relaxation experiments.

## Supplementary Information

Below is the link to the electronic supplementary material.

Supplementary Material 1

## Data Availability

All data supporting the findings of this study are available either within the paper and its Supplementary Information or are available upon request from the authors.
