# Supplementary material for: The perfect carbon TOCSY (iPE TOCSY) facilitates assignment of large biomolecules
Source: J Biomol NMR. 2026 Sep 24;80(1):19. doi: 10.1007/s10858-026-00496-z (PMC13612683; doi:10.1007/s10858-026-00496-z)
Supplement: Supplementary file 1 [file 10858_2026_496_MOESM1_ESM.pdf]

Supplementary Material to:

The perfect carbon TOCSY facilitates assignment of large biomolecules

Arnelle Löbbert[a,b], Aditya Pokharna[a,c], Philip Rößler[a,d], Simon H. Rüdisser[a], Alvar D. Gossert[a,*]

[a] Department of Biology, ETH Zürich, 8093 Zürich, Switzerland

[b] Present address: Novartis Pharma AG, Basel, Switzerland

[c] Present address: MRC Laboratory of Molecular Biology, Cambridge, United Kingdom

[d] Present address: University of Toronto, Toronto, Canada

*Corresponding author: alvar.gossert@biol.ethz.ch

Contents:

Suppl. Figure 1. Comparison of different PE-delays 2

Suppl. Figure 2. Comparison of FLOPSY and DIPSI mixing in the PE-(H)C(CCO)NH-TOCSY 3

Suppl. Figure 3: Pulse sequence of PE-(H)CCH-TOCSY 4

Suppl. Figure 4: Pulse sequence of PE-(H)C(CCO)NH-TOCSY 10

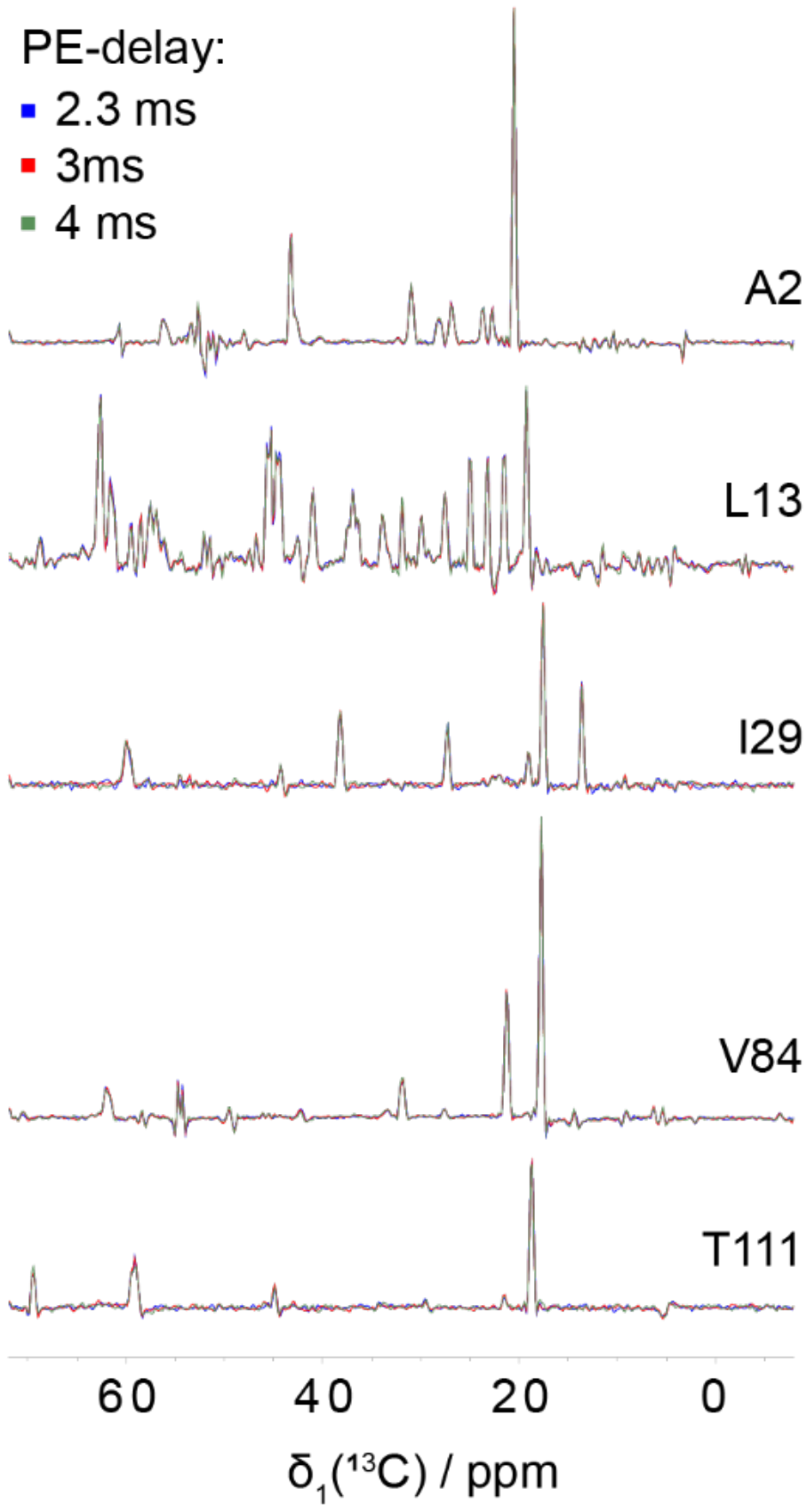

Suppl. Figure 5. Comparison of different PE-delays. 1D slices of one representative of each amino acids carrying a methyl groups are shown. PE-delay of 2.3, 3 and 4 ms (blue, red and green, respectively) were tested within the PE-(H)C(CCO)NH-TOCSY. Experiments were conducted on a 3.8 mM U-^{13}C,^{15}N labeled TM1290 sample over ~4 h at 500 MHz static field. Longer PE-delays did not lead to an increase in the signal intensities. To keep the pulse sequence as condensed as possible, the shortest PE delay was therefore chosen for further experiments.

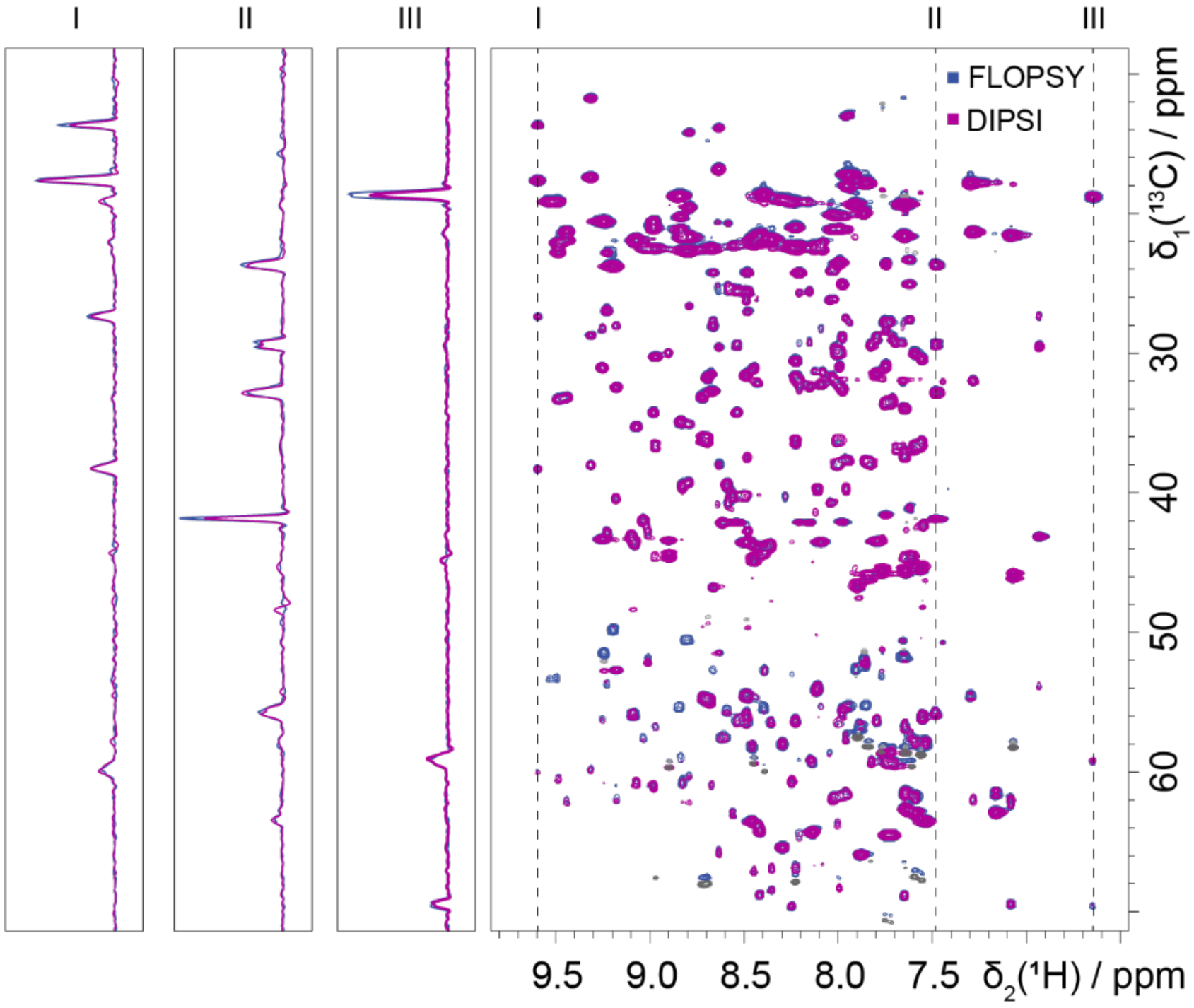

Suppl. Figure 6. 2D [$^1H^N$, ^{13}C-methyl]-planes of the FLOPSY (blue) compared to DIPSI (purple) mixing sequence used in the PE-(H)C(CCO)NH-TOCSY. 1D sliced (marked with a dashed line) of three individual TOCSY towers are shown on the left. The spectra were recorded on a 3.8 mM U-^{13}C,^{15}N labeled TM1290 sample over ~4 h on a 500 MHz spectrometer. The use of a FLOPSY mixing scheme shows a slight enhancement compared to the DIPSI-based sequence and was therefore used in further experiments.

Suppl. Figure 7: Pulse sequence of PE-(H)CCH-TOCSY

```
;HCCH-TOCSY for all aromatic/aliphatic connectivities
;avance-version
;3D sequence with
;   inverse correlation using multiple inept transfer and
;      C-C FLOPSY-16 spinlock
;
;      F1(H,t1) --INEPT--> F2(Cali/aro,t2) --TOCSY--> (Cali'/aro') --INEPT--> F1(H',t3)
;
;phase sensitive (t1)
;phase sensitive (t2)
;high-power TOCSY for transfer between all Cali and Caro (up to 700 MHz with cryoprobe)
;spinlock during second z-filter
;perfect echo for increased resolution and sensitivity on 13C (F2)
;virtual offset (cnst61) for optimal folding
;
;
;(H. Kovacs & A.D. Gossert
;   J. Biomol. NMR  58:101 - 112 (2014))
;
;(L.E. Kay, G.Y. Xu, A.U. Singer, D.R. Muhandiram & J. D. Forman-Kay
;   J. Magn. Reson. B 101: 333 - 337 (1993))
;
;
;$CLASS=HighRes
;$DIM=3D
;$TYPE=
;$SUBTYPE=
;$COMMENT=

prosol relations=<triple>


#include <Avance.incl>
#include <Grad.incl>
#include <Delay.incl>


"p2=p1*2"
"p4=p3*2"
"p22=p21*2"
"d11=30m"
"d12=20u"
"p16=500u"

"d4=1.6m"                     ;tau a

"d22=d21+p4+p2"
"d23=d21-p16-p2/2"
"d24=d22-d21-p2/2-p4/2"
"d25=d22-p4/2-p16"
;"d26=d10+d22*cnst1/4"
;"d27=d26*2+larger(p2,p22)+p3"

"p19=2m"
"p29=300u"
"p30=5m"
"p31=4.4m"

"d16=150u"


"d0=3u"
"d10=3u"

"in0=inf1/2"
"in10=inf2/2"


"DELTA1=d4-p16-d16-4u"
"DELTA2=d4-p16-d16-4u+d0*2+p8"
"DELTA3=d23-p29-d16"
"DELTA4=p22+p2+d10*2+4u"
"DELTA5=d21-p16-d16-4u"
"DELTA6=d4-p16-d16-4u-p3-1.5u+0.5*p1"
"DELTA7=d10*2+larger(p2,p22)+p3/(2*PI)"

"cnst62=((bf2*cnst61)-(o2*1000000))"
"cnst63=-((cnst62*inf2/1000000)*360.0)/2.0"

"d31=(p9*188.448)*l1"    ;FLOPSY
"l1=(d15/(p9*188.448))"
"spoff5=bf2*(cnst21/1000000)-o2"
```

```
"acqt0=-p1*2/PI-4u-1.5u-p3+0.5*p1"

aqseq 312


1 d11 ze
  20m pl12:f2
2 d11 do:f2
  4u BLKGRAD
3 d1
  50u UNBLKGRAD
  d12 pl1:f1 pl3:f3
;------------------------runtime calculations
  "d22=larger(d21,d10)+p4+p2+20u"
  1m
  if "d22 > d32/2"  ; stop increasing PE delay at max value set by user
    {
    20u
    "d22=d32/2"
    }
  1m
  "d23=d21-p16-d16-p2/2"
  "d24=d22-d21-p2/2-p4/2"
  "d25=d22-p4/2-p16-d16"

 ;------------------------start INEPT and evolution on 1H
  (p1 ph3)
  4u
  p16:gp1
  d16
  DELTA1 pl0:f2
  d0
  (p8:sp13 ph1):f2
  d0
  (p2 ph1)
  4u
  p16:gp1
  d16
  DELTA2 pl2:f2
  (p1 ph2)
;------------------------z-filter gradient
  p19:gp3
  d16
;------------------------refocusing INEPT with PE (total length = d22, J(CH) active during d21*2)
  (p3 ph4):f2
  p16:gp2
  d16
  d23
  (p2 ph1):f1
  d24
  (p4 ph6):f2
  p16:gp2
  d16
  d25
  (p3 ph7):f2
;------------------------13C chemical shift evolution
  d10
  (center (p2 ph1):f1 (p22 ph1):f3)
  d10
  (p4 ph8):f2
  DELTA7
  (p3 ph2):f2
;------------------------end of 13C evolution
  4u
  d12 pl15:f2


;------------------------begin FLOPSY16
9 (p9*0.511 ph11):f2
  (p9*1.067 ph12):f2
  (p9*1.822 ph13):f2
  (p9*1.767 ph14):f2
  (p9*1.444 ph15):f2
  (p9*1.767 ph14):f2
  (p9*1.822 ph13):f2
  (p9*1.067 ph12):f2
  (p9*0.511 ph11):f2

  (p9*0.511 ph21):f2
  (p9*1.067 ph22):f2
  (p9*1.822 ph23):f2
  (p9*1.767 ph24):f2
  (p9*1.444 ph25):f2
  (p9*1.767 ph24):f2
```

```
(p9*1.822 ph23):f2
(p9*1.067 ph22):f2
(p9*0.511 ph21):f2

(p9*0.511 ph21):f2
(p9*1.067 ph22):f2
(p9*1.822 ph23):f2
(p9*1.767 ph24):f2
(p9*1.444 ph25):f2
(p9*1.767 ph24):f2
(p9*1.822 ph23):f2
(p9*1.067 ph22):f2
(p9*0.511 ph21):f2

(p9*0.511 ph11):f2
(p9*1.067 ph12):f2
(p9*1.822 ph13):f2
(p9*1.767 ph14):f2
(p9*1.444 ph15):f2
(p9*1.767 ph14):f2
(p9*1.822 ph13):f2
(p9*1.067 ph12):f2
(p9*0.511 ph11):f2

(p9*0.511 ph11):f2
(p9*1.067 ph12):f2
(p9*1.822 ph13):f2
(p9*1.767 ph14):f2
(p9*1.444 ph15):f2
(p9*1.767 ph14):f2
(p9*1.822 ph13):f2
(p9*1.067 ph12):f2
(p9*0.511 ph11):f2

(p9*0.511 ph11):f2
(p9*1.067 ph12):f2
(p9*1.822 ph13):f2
(p9*1.767 ph14):f2
(p9*1.444 ph15):f2
(p9*1.767 ph14):f2
(p9*1.822 ph13):f2
(p9*1.067 ph12):f2
(p9*0.511 ph11):f2

(p9*0.511 ph21):f2
(p9*1.067 ph22):f2
(p9*1.822 ph23):f2
(p9*1.767 ph24):f2
(p9*1.444 ph25):f2
(p9*1.767 ph24):f2
(p9*1.822 ph23):f2
(p9*1.067 ph22):f2
(p9*0.511 ph21):f2

(p9*0.511 ph21):f2
(p9*1.067 ph22):f2
(p9*1.822 ph23):f2
(p9*1.767 ph24):f2
(p9*1.444 ph25):f2
(p9*1.767 ph24):f2
(p9*1.822 ph23):f2
(p9*1.067 ph22):f2
(p9*0.511 ph21):f2

(p9*0.511 ph21):f2
(p9*1.067 ph22):f2
(p9*1.822 ph23):f2
(p9*1.767 ph24):f2
(p9*1.444 ph25):f2
(p9*1.767 ph24):f2
(p9*1.822 ph23):f2
(p9*1.067 ph22):f2
(p9*0.511 ph21):f2

(p9*0.511 ph11):f2
(p9*1.067 ph12):f2
(p9*1.822 ph13):f2
(p9*1.767 ph14):f2
(p9*1.444 ph15):f2
(p9*1.767 ph14):f2
(p9*1.822 ph13):f2
(p9*1.067 ph12):f2
(p9*0.511 ph11):f2

(p9*0.511 ph11):f2
```

```
  (p9*1.067 ph12):f2
  (p9*1.822 ph13):f2
  (p9*1.767 ph14):f2
  (p9*1.444 ph15):f2
  (p9*1.767 ph14):f2
  (p9*1.822 ph13):f2
  (p9*1.067 ph12):f2
  (p9*0.511 ph11):f2

  (p9*0.511 ph21):f2
  (p9*1.067 ph22):f2
  (p9*1.822 ph23):f2
  (p9*1.767 ph24):f2
  (p9*1.444 ph25):f2
  (p9*1.767 ph24):f2
  (p9*1.822 ph23):f2
  (p9*1.067 ph22):f2
  (p9*0.511 ph21):f2

  (p9*0.511 ph21):f2
  (p9*1.067 ph22):f2
  (p9*1.822 ph23):f2
  (p9*1.767 ph24):f2
  (p9*1.444 ph25):f2
  (p9*1.767 ph24):f2
  (p9*1.822 ph23):f2
  (p9*1.067 ph22):f2
  (p9*0.511 ph21):f2

  (p9*0.511 ph21):f2
  (p9*1.067 ph22):f2
  (p9*1.822 ph23):f2
  (p9*1.767 ph24):f2
  (p9*1.444 ph25):f2
  (p9*1.767 ph24):f2
  (p9*1.822 ph23):f2
  (p9*1.067 ph22):f2
  (p9*0.511 ph21):f2

  (p9*0.511 ph11):f2
  (p9*1.067 ph12):f2
  (p9*1.822 ph13):f2
  (p9*1.767 ph14):f2
  (p9*1.444 ph15):f2
  (p9*1.767 ph14):f2
  (p9*1.822 ph13):f2
  (p9*1.067 ph12):f2
  (p9*0.511 ph11):f2

  (p9*0.511 ph11):f2
  (p9*1.067 ph12):f2
  (p9*1.822 ph13):f2
  (p9*1.767 ph14):f2
  (p9*1.444 ph15):f2
  (p9*1.767 ph14):f2
  (p9*1.822 ph13):f2
  (p9*1.067 ph12):f2
  (p9*0.511 ph11):f2
  lo to 9 times l1
;------------------------end FLOPSY16

;------------------------water handling

  d12 pl10:f1
  (p17 ph1)
  (p17*2 ph2)
  4u
  p30:gp4
  d16 pl1:f1
  (p1 ph1)
  4u
  p31:gp4
  d16 pl2:f2
;------------------------reverse INEPT1
  (p3 ph2):f2
  4u
  p16:gp1
  d16
  DELTA5
  (center (p2 ph1) (p4 ph1):f2 )
  4u
  p16:gp1
  d16
  DELTA5
  (center (p1 ph1) (p3 ph1):f2 )
```

```
;------------------------reverse INEPT2
  4u
  p16:gp1
  d16
  DELTA1
  (center (p2 ph1) (p8:sp13 ph1):f2 )
  4u
  p16:gp1
  d16
  DELTA6 pl2:f2
  (center (p1 ph1) (p3 ph1 3u p3 ph5):f2 )
  4u  pl12:f2
;------------------------start acquisition
  go=2 ph31 cpd2:f2
  d11 do:f2 mc #0 to 2
     F1PH(calph(ph3, +90), caldel(d0, +in0))
     F2PH(calph(ph4, +90) & calph(ph6, +90) & calph(ph7, +90) & calph(ph8,+90), caldel(d10, +in10) & calph(ph8, cnst63))
exit


ph1=0
ph2=1
ph3=0 2
ph4=0 0 2 2
ph5=0 0 0 0
ph6=1 1
ph7=0
ph8=1
ph9=3
ph10=0

ph11=(720) 0
ph12=(720) 90
ph13=(720) 135
ph14=(720) 630
ph15=(720) 45

ph21=(720) 360
ph22=(720) 450
ph23=(720) 495
ph24=(720) 270
ph25=(720) 405

ph31=0 2 2 0


;pl0 : 120dB
;pl1 : f1 channel - power level for pulse (default)
;pl2 : f2 channel - power level for pulse (default)
;pl3 : f3 channel - power level for pulse (default)
;pl10: f1 channel - power level for TOCSY-spinlock (trim pulse)
;pl12: f2 channel - power level for CPD/BB decoupling
;pl15: f2 channel - power level for TOCSY-spinlock (careful!)
;sp5: f2 channel - shaped pulse 180 degree   (C=O off resonance)
;p1 : f1 channel -  90 degree high power pulse
;p2 : f1 channel - 180 degree high power pulse
;p3 : f2 channel -  90 degree high power pulse
;p4 : f2 channel - 180 degree high power pulse
;p9 : f2 channel -  90 degree low power pulse          [15-20 usec]
;p14: f2 channel - 180 degree shaped pulse
;p16: homospoil/gradient pulse                         [500 usec]
;p17: f1 channel - trim pulse                          [2.5 msec]
;p19: gradient pulse 2                                 [2 msec]
;p22: f3 channel - 180 degree high power pulse
;p29: gradient pulse 3                                 [300 usec]
;p30: gradient pulse 4                                 [5 msec]
;p31: gradient pulse 5                                 [4.4 msec]
;d0 : incremented delay (F1 in 3D)                     [3 usec]
;d1 : relaxation delay; 1-5 * T1 !!! USE >= 2.0 s FOR STRONG PL15
;d4 : 1/(4J(CH)) - tau a                               [1.6 msec]
;d10: incremented delay (F2 in 3D)                     [3 usec]
;d11: delay for disk I/O                               [30 msec]
;d12: delay for power switching                        [20 usec]
;d15: flopsy16 mixing time                             [15-25 msec]
;d16: delay for homospoil/gradient recovery
;d21: 1/(6J'(CH))                                      [1.1 msec]
;d22: PE delay                                                                                             [2.3 msec]
;d31: length of DIPSI-3 cycle as executed = (p9*54.33*4)*l1
;d32: maximal length of PE delay [2.3 - 6 ms]
;cnst1: PE (small proteins set to 1 = full PE, large proteins set to 0)
;cnst21: CO chemical shift (offset, in ppm)            [174 ppm]
;cnst23: Caliphatic chemical shift (offset, in ppm)
;cnst61: virtual chemical shift offset (e.g. 42 ppm)
;o2p: Caliphatic chemical shift (cnst23)
;;l1: loop for FLOPSY16 cycle: ((p6*188.448) * l1) = mixing time
```

```
;;l1: loop for DIPSI3 cycle:
;       mixing time = ((p9*54.33*4) * l1)              [12 msec]
;inf1: 1/SW(Hali) = 2 * DW(Hali)
;inf2: 1/SW(C) = 2 * DW(C)
;in0: 1/(2 * SW(Hali)) = DW(Hali)
;nd0: 2
;in10: 1/(2 * SW(C)) = DW(C)
;nd10: 2
;ns: 8 * n
;ds: 32
;td1: number of experiments in F1
;td2: number of experiments in F2
;FnMODE: States-TPPI in F1 (not suitable for TPPI)
;FnMODE: States-TPPI (or TPPI) in F2
;cpd2: decoupling according to sequence defined by cpdprg2
;pcpd2: f2 channel - 90 degree pulse for decoupling sequence


;use gradient ratio:    gp 1 : gp 2 : gp 3 : gp 4
;                         16 :   16 :   30 :   60

;for z-only gradients:
;gpz1: 16%
;gpz2: 16%
;gpz3: 30%
;gpz4: 60%

;use gradient files:
;gpnam1: SMSQ10.50
;gpnam2: SMSQ10.50
;gpnam3: SMSQ10.100
;gpnam4: SMSQ10.100
```

Suppl. Figure 8: Pulse sequence of PE-(H)C(CCO)NH-TOCSY

```
;hccconhgpwg3d3 shortened version
;avance-version
;HCCCONH 3D sequence with inverse correlation for triple resonance using flopsy and inept transfer steps
;
;
;F1(H) -> F2(C,t1 ) -> F2(C=O) -> F3(N,t2) -> F1(H,t3)

;
;
;on/off resonance Ca and C=O pulses using shaped pulse
;phase sensitive (t1)
;phase sensitive (t2)
;using constant time in t2
;water suppression using watergate sequence
;(use parameterset HCCCONHGPWG3D3)
;
;G.T. Montelione, B.A. Lyons, S.D. Emerson & M. Tashiro,
;   J. Am. Chem. Soc. 114, 10974-75 (1992)
;S. Grzesiek, J. Anglister & A. Bax, J. Magn. Reson. 101 B, 114-9 (1993)
;B.A. Lyons & G.T. Montelione, J. Magn. Reson. 101 B, 206-9 (1993)
;T.M. Logan, E.T. Olejniczak, R.X. Xu & S.W. Fesik,
;   J. Biomol. NMR 3, 225-31 (1993)
;R.T. Clowes, W. Boucher, C.H. Hardman, P.J. Domaille & E.D. Laue,
;   J. Biomol. NMR 3, 349-354 (1993)
;T. Carlomagno, M. Maurer, M. Sattler, M.G. Schwendinger, S.J. Glaser
;   & C. Griesinger, J. Biomol. NMR 8, 161-170 (1996)
;
;$CLASS=HighRes
;$DIM=3D
;$TYPE=
;$SUBTYPE=
;$COMMENT=


prosol relations=<triple>


#include<Avance.incl>
#include<Grad.incl>
#include<Delay.incl>


"p2=p1*2"
"p22=p21*2"
"d11=30m"
"d12=20u"

"d3=1.1m"
"d31=d3/2-p14/2-p22-d0"
"d32=d3/2-p14/2-p2-d0"
"d4=1/(cnst4*4)"
"d41=1/(cnst4*cnst41)"
"d21=3.6m"
"d22=4.4m"
"d23=12.4m"
"d25=5.5m"
"d26=2.3m"

"d42=d41+p4+p2"
"d43=d41-p19-d16-p2/2"
"d44=d42-d41-p2/2-p4/2"
"d45=d42-p4/2-p19-d16"

"d0=3u"
"d10=d23/2-p14/2"
"d29=d23/2-p14/2-p26-d25-4u"
"d30=d23/2-p14/2"

"in0=inf1/2"
"in10=inf2/4"

"in29=in10"
"in30=in10"

#ifdef FLOPSY
"l1=(d15/(p9*188.448))" ;FLOPSY
"d61=(p9*188.448)*l1"
#else
"l1=(d15/(p9*115.112))+0.5" ;DIPSY
```

```
"d61=(p9*115.112)*l1"
#endif


"DELTA1=d3/2-p14/2-16u"
"DELTA2=d23"
"DELTA3=d26-p16-d16-p11-12u"
"DELTA7=d0*2+larger(p2,p22)+p3/(2*PI)"


"spoff2=0"
"spoff3=0"
"spoff5=bf2*((cnst21-cnst23)/1000000)"
"spoff7=bf2*((cnst22-cnst21)/1000000)"
"spoff8=0"
"spoff18=bf2*((cnst25-cnst23)/1000000)"
;"spoff9=bf2*((cnst22-cnst23)/1000000)"


aqseq 321


1 ze
  d61
  d11 pl16:f3
2 d11 do:f3

;-------------------------------- runtime calculations
    "d42=larger(d41,d0)+p4+p2+20u"
    1m
    if "d42 > d40/2"  ; stop increasing PE delay at max value set by user
      {
      20u
      "d42=d40/2"
      }
    1m
    "d43=d41-p19-d16-p2/2"
    "d44=d42-d41-p2/2-p4/2"
    "d45=d42-p4/2-p19-d16"
;-------------------------------- end runtime calculations

;-------------------------------- start at aliphatic/aromatic frequency
3 d11 fq=cnst23(bf ppm):f2
  d1


  50u UNBLKGRAD
  d12 pl1:f1 pl2:f2 pl3:f3
;-------------------------------- INEPT 1H->13C

  (p1 ph3):f1
;  d41
;  (center (p2 ph1):f1 (p15:sp18 ph1):f2 )
;  d41 pl2:f2
  d4
  (center (p2 ph1):f1 (p4 ph1):f2 )
  d4
  (p1 ph2):f1
;-------------------------------- z-filter gradient
  4u
  p16:gp1
  d16


#ifdef PE
;-------------------------------- refocusing INEPT with PE (total length = d42, J(CH) active during d41*2)
  (p3 ph4):f2
  p19:gp2
  d16
  d43
  (p2 ph1):f1
  d44
  (p4 ph16):f2
  p19:gp2
  d16
  d45
  (p3 ph17):f2
;-------------------------------- 13C chemical shift evolution
  d0
  (center (p2 ph1):f1 (p22 ph1):f3)
  d0
  (p4 ph18):f2
  DELTA7 fq=cnst24(bf ppm):f2 ;change to offset for spinlock
;-------------------------------- end of 13C evolution
```

```
#else
;-------------------------------- regular refocusing INEPT and evolution on 13C

  (p3 ph4):f2
  d0
  (p22 ph1):f3
  d31
  (p14:sp5 ph1):f2  ; carbonyl off-resonance refocussing
  d32 pl2:f2
  (p2 ph1):f1
  d0
  (p4 ph1):f2
  16u
  DELTA1
  (p14:sp5 ph1):f2   ; carbonyl off-resonance refocussing
  DELTA1
  12u  fq=cnst24(bf ppm):f2 ;change to offset for spinlock
 4u pl2:f2
;-------------------------------- end regular refocusing INEPT and evolution on 13C
#endif

;-------------------------------- begin TOCSY (choose FLOPSY or DIPSI with ZGOPTNS)

#ifdef FLOPSY                 ;begin FLOPSY16

  (p3 ph1):f2
  2u
  4u pl15:f2

7 (p9*0.511 ph11):f2
  (p9*1.067 ph12):f2
  (p9*1.822 ph13):f2
  (p9*1.767 ph14):f2
  (p9*1.444 ph15):f2
  (p9*1.767 ph14):f2
  (p9*1.822 ph13):f2
  (p9*1.067 ph12):f2
  (p9*0.511 ph11):f2

  (p9*0.511 ph21):f2
  (p9*1.067 ph22):f2
  (p9*1.822 ph23):f2
  (p9*1.767 ph24):f2
  (p9*1.444 ph25):f2
  (p9*1.767 ph24):f2
  (p9*1.822 ph23):f2
  (p9*1.067 ph22):f2
  (p9*0.511 ph21):f2

  (p9*0.511 ph21):f2
  (p9*1.067 ph22):f2
  (p9*1.822 ph23):f2
  (p9*1.767 ph24):f2
  (p9*1.444 ph25):f2
  (p9*1.767 ph24):f2
  (p9*1.822 ph23):f2
  (p9*1.067 ph22):f2
  (p9*0.511 ph21):f2

  (p9*0.511 ph11):f2
  (p9*1.067 ph12):f2
  (p9*1.822 ph13):f2
  (p9*1.767 ph14):f2
  (p9*1.444 ph15):f2
  (p9*1.767 ph14):f2
  (p9*1.822 ph13):f2
  (p9*1.067 ph12):f2
  (p9*0.511 ph11):f2

  (p9*0.511 ph11):f2
  (p9*1.067 ph12):f2
  (p9*1.822 ph13):f2
  (p9*1.767 ph14):f2
  (p9*1.444 ph15):f2
  (p9*1.767 ph14):f2
  (p9*1.822 ph13):f2
  (p9*1.067 ph12):f2
  (p9*0.511 ph11):f2

  (p9*0.511 ph11):f2
  (p9*1.067 ph12):f2
  (p9*1.822 ph13):f2
  (p9*1.767 ph14):f2
```

```
(p9*1.444 ph15):f2
(p9*1.767 ph14):f2
(p9*1.822 ph13):f2
(p9*1.067 ph12):f2
(p9*0.511 ph11):f2

(p9*0.511 ph21):f2
(p9*1.067 ph22):f2
(p9*1.822 ph23):f2
(p9*1.767 ph24):f2
(p9*1.444 ph25):f2
(p9*1.767 ph24):f2
(p9*1.822 ph23):f2
(p9*1.067 ph22):f2
(p9*0.511 ph21):f2

(p9*0.511 ph21):f2
(p9*1.067 ph22):f2
(p9*1.822 ph23):f2
(p9*1.767 ph24):f2
(p9*1.444 ph25):f2
(p9*1.767 ph24):f2
(p9*1.822 ph23):f2
(p9*1.067 ph22):f2
(p9*0.511 ph21):f2

(p9*0.511 ph21):f2
(p9*1.067 ph22):f2
(p9*1.822 ph23):f2
(p9*1.767 ph24):f2
(p9*1.444 ph25):f2
(p9*1.767 ph24):f2
(p9*1.822 ph23):f2
(p9*1.067 ph22):f2
(p9*0.511 ph21):f2

(p9*0.511 ph11):f2
(p9*1.067 ph12):f2
(p9*1.822 ph13):f2
(p9*1.767 ph14):f2
(p9*1.444 ph15):f2
(p9*1.767 ph14):f2
(p9*1.822 ph13):f2
(p9*1.067 ph12):f2
(p9*0.511 ph11):f2

(p9*0.511 ph11):f2
(p9*1.067 ph12):f2
(p9*1.822 ph13):f2
(p9*1.767 ph14):f2
(p9*1.444 ph15):f2
(p9*1.767 ph14):f2
(p9*1.822 ph13):f2
(p9*1.067 ph12):f2
(p9*0.511 ph11):f2

(p9*0.511 ph21):f2
(p9*1.067 ph22):f2
(p9*1.822 ph23):f2
(p9*1.767 ph24):f2
(p9*1.444 ph25):f2
(p9*1.767 ph24):f2
(p9*1.822 ph23):f2
(p9*1.067 ph22):f2
(p9*0.511 ph21):f2

(p9*0.511 ph21):f2
(p9*1.067 ph22):f2
(p9*1.822 ph23):f2
(p9*1.767 ph24):f2
(p9*1.444 ph25):f2
(p9*1.767 ph24):f2
(p9*1.822 ph23):f2
(p9*1.067 ph22):f2
(p9*0.511 ph21):f2

(p9*0.511 ph21):f2
(p9*1.067 ph22):f2
(p9*1.822 ph23):f2
(p9*1.767 ph24):f2
(p9*1.444 ph25):f2
(p9*1.767 ph24):f2
(p9*1.822 ph23):f2
(p9*1.067 ph22):f2
(p9*0.511 ph21):f2
```

```
  (p9*0.511 ph11):f2
  (p9*1.067 ph12):f2
  (p9*1.822 ph13):f2
  (p9*1.767 ph14):f2
  (p9*1.444 ph15):f2
  (p9*1.767 ph14):f2
  (p9*1.822 ph13):f2
  (p9*1.067 ph12):f2
  (p9*0.511 ph11):f2

  (p9*0.511 ph11):f2
  (p9*1.067 ph12):f2
  (p9*1.822 ph13):f2
  (p9*1.767 ph14):f2
  (p9*1.444 ph15):f2
  (p9*1.767 ph14):f2
  (p9*1.822 ph13):f2
  (p9*1.067 ph12):f2
  (p9*0.511 ph11):f2
  lo to 7 times l1
;-------------------------------- end FLOPSY

#else                                            ;begin DIPSI2

  (p3 ph2):f2
  2u
  4u pl15:f2

9 (p9*3.556 ph23):f2
  (p9*4.556 ph25):f2
  (p9*3.222 ph23):f2
  (p9*3.167 ph25):f2
  (p9*0.333 ph23):f2
  (p9*2.722 ph25):f2
  (p9*4.167 ph23):f2
  (p9*2.944 ph25):f2
  (p9*4.111 ph23):f2

  (p9*3.556 ph25):f2
  (p9*4.556 ph23):f2
  (p9*3.222 ph25):f2
  (p9*3.167 ph23):f2
  (p9*0.333 ph25):f2
  (p9*2.722 ph23):f2
  (p9*4.167 ph25):f2
  (p9*2.944 ph23):f2
  (p9*4.111 ph25):f2

  (p9*3.556 ph25):f2
  (p9*4.556 ph23):f2
  (p9*3.222 ph25):f2
  (p9*3.167 ph23):f2
  (p9*0.333 ph25):f2
  (p9*2.722 ph23):f2
  (p9*4.167 ph25):f2
  (p9*2.944 ph23):f2
  (p9*4.111 ph25):f2

  (p9*3.556 ph23):f2
  (p9*4.556 ph25):f2
  (p9*3.222 ph23):f2
  (p9*3.167 ph25):f2
  (p9*0.333 ph23):f2
  (p9*2.722 ph25):f2
  (p9*4.167 ph23):f2
  (p9*2.944 ph25):f2
  (p9*4.111 ph23):f2
  lo to 9 times l1  ;end DIPSI2
;-------------------------------- end DIPSI
#endif

  d12 pl19:f1 ;pl1:f2
  (p26 ph1):f1
  d12 cpds1:f1 ph2

;-------------------------------- change to carbonyl frequency
  4u
  d12 fq=cnst21(bf ppm):f2 ; carbonyl frequency

;-------------------------------- CO -> N INEPT
#ifdef FLOPSY
  (p13:sp2 ph2):f2 ; carbonyl on-resonance 90
#else
  (p13:sp2 ph1):f2 ; carbonyl on-resonance 90
```

```
#endif
  DELTA2 pl0:f2 pl3:f3
  (center (p14:sp3 ph1):f2 (p22 ph8):f3 ) ; carbonyl on-resonance refocussing
  DELTA2
  (p13:sp8 ph1):f2 ; carbonyl on-resonance 90

  4u
  4u do:f1
  (p26 ph10):f1
  4u
;-------------------------------- z-filter
  p16:gp2
  d16

;-------------------------------- CO -> N refocussing INEPT with N evolution
  (p26 ph2):f1
  4u cpds1:f1 ph1

  (p21 ph6):f3
  d30
  (p14:sp7 ph1):f2 ; Calpha off-resonance refocussing
  d30
  (center (p14:sp3 ph1):f2 (p22 ph1):f3 )
  d10
  (p14:sp7 ph1):f2  ; Calpha off-resonance refocussing
  d29
  4u do:f1
  (p26 ph9):f1
  d25
  (p21 ph1):f3

;-------------------------------- z-filter
  p16:gp3
  d16 pl0:f1

;-------------------------------- water flip-down
  (p11:sp1 ph7):f1
  4u
  4u pl1:f1
;-------------------------------- N -> H refocussing INEPT with soft watergate

  (p1 ph1)
  4u
  p16:gp4
  d16
  DELTA3 pl0:f1
  (p11:sp1 ph7):f1
  4u
  4u pl1:f1
  (center (p2 ph1) (p22 ph1):f3 )
  4u pl0:f1
  (p11:sp1 ph7):f1
  4u
  DELTA3
  p16:gp4
  d16 pl16:f3
  4u BLKGRAD
;-------------------------------- acquisition
  go=2 ph31 cpd3:f3
  d11 do:f3 mc #0 to 2

#ifdef PE
    F1PH(calph(ph4, +90) & calph(ph16, +90) & calph(ph17, +90) & calph(ph18,+90), caldel(d0, +in0))
#else
    F1PH(calph(ph4, +90), caldel(d0, +in0))
#endif
    F2PH(calph(ph6, -90), caldel(d10, +in10) & caldel(d29, +in29) & caldel(d30, -in30))


 exit

;-------------------------------- phases

ph1=0
ph2=1
ph4=0 0 0 0 2 2 2 2
ph3=0 0 2 2
ph6=0 2
ph7=2
ph8=0
ph9=3
ph10=2
ph16=1
ph17=0
ph18=1
```

```
ph31=0 2 2 0 2 0 0 2

#ifdef FLOPSY
ph11=(720) 0
ph12=(720) 90
ph13=(720) 135
ph14=(720) 630
ph15=(720) 45

ph21=(720) 360
ph22=(720) 450
ph23=(720) 495
ph24=(720) 270
ph25=(720) 405
#else
ph23=0
ph25=2
#endif

;pl0 : 120dB
;pl1 : f1 channel - power level for pulse (default)
;pl3 : f3 channel - power level for pulse (default)
;pl15: f2 channel - power level for TOCSY-spinlock
;pl16: f3 channel - power level for CPD/BB decoupling
;pl19: f1 channel - power level for CPD/BB decoupling
;sp1: f1 channel - shaped pulse  90 degree  (H2O on resonance)
;sp2: f2 channel - shaped pulse  90 degree  (C=O on resonance)
;sp3: f2 channel - shaped pulse 180 degree  (on resonance)
;sp5: f2 channel - shaped pulse 180 degree  (C=O off resonance)
;sp7: f2 channel - shaped pulse 180 degree  (Ca off resonance)
;sp8: f2 channel - shaped pulse  90 degree  (on resonance)
;                  for time reversed pulse
;sp9: f2 channel - shaped pulse 180 degree  (Ca on resonance)
;     sp9 might require higher selectivity than sp3
;p1 : f1 channel -  90 degree high power pulse
;p2 : f1 channel - 180 degree high power pulse
;p9 : f2 channel -  90 degree low power pulse
;p11: f1 channel -  90 degree shaped pulse            [1 msec]
;p13: f2 channel -  90 degree shaped pulse
;p14: f2 channel - 180 degree shaped pulse
;p16: homospoil/gradient pulse                        [1 msec]
;p21: f3 channel -  90 degree high power pulse
;p22: f3 channel - 180 degree high power pulse
;p24: f2 channel - 180 degree shaped pulse (sp9)
;p26: f1 channel -  90 degree pulse at pl19
;d0 : incremented delay (F1 in 3D)                    [3 usec]
;d1 : relaxation delay; 1-5 * T1
;d4:  1/(4 * J(CH)                                       [1.7 msec]
;d41: 1/(6 * J(CH) [1.1 msec] or 1/(8 * J(CH) [0.8 msec] for methyls
;d10: incremented delay (F2 in 3D) = d23/2-p14/2
;d11: delay for disk I/O                              [30 msec]
;d12: delay for power switching                       [20 usec]
;d15: TOCSY mixing time                               [20-30 msec]
;d16: delay for homospoil/gradient recovery
;d21: 1/(2J(CaCO))                                    [3.6 msec]
;d22: 1/(2J'(CaCO)                                    [4.4 msec]
;d23: constant time delay T(N) = 1/(4J'(NCO)          [12.4 msec]
;d25: 1/(2J'(NH))                                     [5.5 msec]
;d26: 1/(4J(NH))                                      [2.3 msec]
;d29: incremented delay (F2 in 3D) = d23/2-p14/2-p26-d25-4u
;d30: decremented delay (F2 in 3D) = d23/2-p14/2
;d42: PE delay                                         [2.3 msec]
;d40: maximal length of PE delay                      [2.3 - 6 ms]
;d61: effective TOCSY spin lock duration
;cnst4: J(CH) coupling                                [120-160 Hz]
;cnst41: CHx Multiplicity factor (CH: 4, CH2: 6, CH3: 8)
;cnst21: CO chemical shift (offset, in ppm)
;cnst22: Calpha chemical shift (offset, in ppm)
;cnst23: Caliphatic chemical shift (offset, in ppm)
;o2p: Caliphatic chemical shift (cnst23)
;cnst24: spinlock frequency
;cnst25: 70ppm adiabatic matched sweep pulse
;l1: loop count for DIPSI or FLOPSY cycle: ((p6*115.112) * l1) = mixing time
;inf1: 1/SW(Cali) = 2 * DW(Cali)
;inf2: 1/SW(N) = 2 * DW(N)
;in0: 1/(2 * SW(Cali)) =  DW(Cali)
;nd0: 2
;in10: 1/(2 * SW(N)) = DW(N)
;nd10: 4
;in29: = in10
;in30: = in10
;NS: 16 * n
;DS: >= 16
;td1: number of experiments in F1
```

```
;td2: number of experiments in F2       td2 max = 2 * d30 / in30
;FnMODE: States-TPPI (or TPPI) in F1
;FnMODE: States-TPPI (or TPPI) in F2
;cpds1: decoupling according to sequence defined by cpdprg1
;cpd3: decoupling according to sequence defined by cpdprg3
;pcpd1: f1 channel - 90 degree pulse for decoupling sequence
;pcpd3: f3 channel - 90 degree pulse for decoupling sequence


;use gradient ratio:    gp 1 : gp 2 : gp 3 : gp 4
;                         50 :   40 :   60 :   30

;for z-only gradients:
;gpz1: 50%
;gpz2: 40%
;gpz3: 60%
;gpz4: 30%

;use gradient files:
;gpnam1: SINE.100
;gpnam2: SINE.100
;gpnam3: SINE.100
;gpnam4: SINE.100
```
